# Assessment of comprehensive HIV/AIDS knowledge level among in-school adolescents in eastern Ethiopia

**DOI:** 10.7448/IAS.16.1.17349

**Published:** 2013-03-20

**Authors:** Lemessa Oljira, Yemane Berhane, Alemayehu Worku

**Affiliations:** 1 College of Health Sciences, Department of Public Health, Haramaya University, Harar, Ethiopia; 2 Addis Continental Institute of Public Health, Department of Epidemiology, Addis Ababa, Ethiopia; 3 School of Public Health, Department of Biostatistics, Addis Ababa University, Addis Ababa, Ethiopia

**Keywords:** in-school, adolescents, HIV/AIDS, comprehensive knowledge

## Abstract

**Introduction:**

In Ethiopia, more adolescents are in school today than ever before; however, there are no studies that have assessed their comprehensive knowledge of HIV/AIDS. Thus, this study tried to assess the level of this knowledge and the factors associated with it among in-school adolescents in eastern Ethiopia.

**Methods:**

A cross-sectional school-based study was conducted using a facilitator-guided self-administered questionnaire. The respondents were students attending regular school in 14 high schools located in 14 different districts in eastern Ethiopia. The proportion of in-school adolescents with comprehensive HIV/AIDS knowledge was computed and compared by sex. The factors that were associated with the comprehensive HIV/AIDS knowledge were assessed using bivariate and multivariable logistic regression.

**Results:**

Only about one in four, 677 (24.5%), in-school adolescents have comprehensive HIV/AIDS knowledge. The knowledge was better among in-school adolescents from families with a relatively middle or high wealth index (adjusted OR [95% CI]=1.39 [1.03–1.87] and 1.75 [1.24–2.48], respectively), who got HIV/AIDS information mainly from friends or mass media (adjusted OR [95% CI]=1.63 [1.17–2.27] and 1.55 [1.14–2.11], respectively) and who received education on HIV/AIDS and sexual matters at school (adjusted OR [95% CI]=1.59 [1.22–2.08]). The females were less likely to have comprehensive HIV/AIDS knowledge compared to males (adjusted OR and [95% CI]=0.60 [0.49–0.75]).

**Conclusions:**

In general, only about a quarter of in-school adolescents had comprehensive HIV/AIDS knowledge. Although the female adolescents are highly vulnerable to HIV infection and its effects, they were by far less likely to have comprehensive HIV/AIDS knowledge. HIV/AIDS information, education and communication activities need to be intensified in high schools.

## Introduction

In Ethiopia, a large number of adolescents are enrolled in high schools, and a significant proportion of rural students attend high school away from their home village. The level of comprehensive HIV/AIDS knowledge and access to HIV/AIDS information and services have been matters of great concern [1]. In Ethiopia, an awareness of HIV/AIDS among adult population has been found to be 97.6% for men and 96.2% for women, while the knowledge of preventive strategies is estimated to be 62.0% for men and 48.7% for women. The levels of overall (57%) and comprehensive (18.5%) knowledge of HIV/AIDS among different population groups including adolescents were lower [2,3]. Similarly, the comprehensive knowledge of modes of HIV transmission of in-school adolescents was lower than that of the general awareness or the separate modes of transmission [4,5]. Studies from other African countries and eastern India also revealed that comprehensive knowledge of HIV/AIDS ranged from 9% to 42% [6–8]; however, studies from Brazil and Europe showed a higher (more than 90%) degree of HIV/AIDS and related issues awareness [9,10].

Previous studies conducted in Ethiopia revealed that residing in urban areas, higher educational attainment and male gender are positively associated with increased awareness of HIV prevention methods [2]. Studies from other countries have also found out that comprehensive HIV/AIDS knowledge is associated with communication with guardians or parents and peers about sexual topics, while living in poor households and disadvantaged neighbourhoods is associated with inaccurate knowledge of the transmission and prevention methods of HIV [6,8].

In Ethiopia, there are only a few studies that have assessed the level of the comprehensive HIV/AIDS knowledge of in-school adolescents. The available studies revealed that sexual debut during adolescence is associated with the risk of being HIV positive at later ages and that secondary school adolescents have the highest HIV positive proportion among the youth age groups in Ethiopia [5,11]. Furthermore, after three decades of AIDS pandemic, it is believed that measuring knowledge of HIV/AIDS by a single awareness question (asking a question such as “Have you ever heard of HIV/AIDS?”) is simply misleading and inappropriate. This study tried to assess the level of the comprehensive HIV/AIDS knowledge and the factors associated with it among in-school adolescents in eastern Ethiopia.

## Methods

The study design was a cross-sectional school-based survey with internal comparison. The study was conducted in eastern Ethiopia, and it involved 14 randomly selected high schools found in 14 different districts. The sample size (*N*=2860) was determined by OpenEpi web-based epidemiological calculator based on the assumptions of 95% significance level; 25% males and 19% females had the outcome [3], considering 3:1 male–female proportion. All the students who were attending regular classes in the selected high schools were eligible for the study, and the respondents were randomly selected by the 3:1 male–female proportion (72% male and 28% female) based on the enrolment data for that academic year. Data were collected by a facilitator-guided self-administered structured questionnaire adapted from WHO sexual and reproductive health questionnaires [12]. In each school, students who were selected for the study were summoned by gender to designated classrooms, and data were collected simultaneously to overcome information contamination. The data collection process was facilitated by gender-matched facilitators. Facilitators were university lecturers who received training on the study procedures and spoke the local language fluently. Two facilitators per classroom were assigned to facilitate the data collection process. To check its consistency, the questionnaire was prepared in English and translated into Afan Oromo and Amharic and then back to English by independent bilingual language experts.

The dependent variable was comprehensive HIV/AIDS knowledge measured by correct answers to HIV/AIDS diagnosis and treatment, HIV transmission modes and HIV prevention methods; comprehensive knowledge was then redefined by the ability to identify correctly at least two major ways of preventing sexual transmission of HIV, to reject at least two most common local misconceptions about HIV transmission and by the correct knowledge of HIV diagnosis method. The independent variables were sex, age, area of residence, wealth index, parents’ vital status, father's educational status, mother's educational status, major source of HIV/AIDS information, discussion on sexual topics with parents or other family members and ever having been taught HIV/AIDS and related issues at school.

Data were double-entered onto the EPI-data Version 3.1 software by defining legal values for each variable and setting skip patterns. The double-entered data were verified and the cleaned version was exported to Stata/SE 11.0 software for analysis. The level of knowledge was computed and compared for males and females. The factors associated with comprehensive HIV/AIDS knowledge at bivariate were identified, and the variables with *P* value of 0.25 and less were taken to multivariable analysis. The model was built with backward elimination.

The study was conducted after obtaining approval from the Institutional Review Committee of Haramaya University and the necessary permission from other concerned educational authorities. The confidentiality of the information was maintained by excluding personal identifiers, and data were collected after getting informed consent and/or assent from the teacher–parent joint committee and/or every respondent.

## Results

Of the 2860 students invited to fill out the facilitator-guided self-administered questionnaire, 2766 students responded adequately, making the response rate 96.7%. The majority, 1985 (71.8%), of the respondents were male. The majority of in-school students, 1901 (68.7%), had families in rural areas (Table 1). The age of the respondents ranged from 14 to 19 years, and the mean was 17.1. Only about one in four, 677 (24.5%), in-school adolescents had comprehensive HIV/AIDS knowledge, while the males had more comprehensive HIV/AIDS knowledge (27.3%) compared to the females (17.3%) (*P*<0.001). The combined comprehensive HIV/AIDS and pregnancy knowledge was very low, 139 (5%). However, the males were more likely to have the combined comprehensive knowledge (5.7%) compared to the females (3.2%) (*P*=0.006) (Table 2).

**Table 1:** Background characteristics of in-school adolescents and their families, Eastern Hararge Zone, Oromia Regional State, Eastern Ethiopia, 2011

Variables	Category	Number	%
Family residence	Rural	1901	68.7
	Urban	865	31.3
Respondent's sex	Male	1985	71.8
	Female	781	28.2
Age group	<18 years	1141	41.3
	≥18 years	1625	58.7
Family wealth index	Low	337	12.2
	Middle	1954	70.6
	High	475	17.2
Parents’ vital status	Both dead	88	3.2
	One alive	565	20.4
	Both alive	2113	76.4
Father's educational status	No education	1374	60.6
	Primary	623	27.9
	Secondary	180	7.9
	12 plus	89	4.0
Mother's educational status	No education	1807	71.6
	Primary	581	23.0
	Secondary	101	4.0
	12 plus	36	1.4

**Table 2:** Comprehensive HIV/AIDS and pregnancy knowledge by gender among in-school adolescents, Eastern Hararge Zone, Oromia Regional State, Eastern Ethiopia, 2011

Variable	Male [*n* (%)=1995 (71.8)]	Female [*n* (%)=781 (28.2)]	Total *n* (%)
Comprehensive knowledge of HIV diagnosis method			
Yes	1223 (61.6)	392 (50.2)	1615 (58.4)
No	762 (38.4)	389 (49.8)	1151 (41.6)
Comprehensive knowledge of HIV transmission modes			
Yes	1425 (71.8)	466 (59.7)	1891 (68.4)
No	560 (28.2)	315 (40.3)	875 (31.6)
Comprehensive knowledge of HIV prevention methods			
Yes	1136 (57.2)	376 (48.1)	1512 (54.7)
No	849 (42.8)	405 (51.9)	1254 (45.3)
Comprehensive HIV/AIDS knowledge			
Yes	542 (27.3)	135 (17.3)	677 (24.5)
No	1443 (72.7)	646 (82.7)	2089 (75.5)
Comprehensive knowledge of pregnancy occurrence dates in relation to menstrual cycle			
Yes	681 (34.3)	347 (44.4)	1028 (37.2)
No	1304 (65.7)	434 (55.6)	1738 (62.8)
Comprehensive knowledge of some pregnancy prevention methods			
Yes	1086 (54.7)	228 (29.2)	1314 (47.5)
No	899 (45.3)	553 (70.8)	1452 (52.5)
Comprehensive pregnancy knowledge			
Yes	403 (20.3)	109 (14.0)	512 (18.5)
No	1582 (79.7)	672 (86.0)	2254 (81.5)
Comprehensive knowledge of HIV/AIDS and pregnancy			
Yes	114 (5.7)	25 (3.2)	139 (5.0)
No	1871 (94.3)	756 (96.8)	2627 (95.0)

### Predictors of comprehensive HIV/AIDS knowledge

The logistic regression showed that the females were 40% less likely to have comprehensive HIV/AIDS knowledge compared to the males (adjusted OR [95% CI]=0.60 [0.49–0.75]). Family wealth index was associated with comprehensive HIV/AIDS knowledge, in that adolescents from a middle or high family wealth index were more likely to have comprehensive HIV/AIDS knowledge compared to those from a low family wealth index (adjusted OR [95% CI]=1.39 [1.03–1.87] and 1.75 [1.24–2.47], respectively). The family wealth index effect was stronger and significant for adolescents from families in rural areas compared to those from families in urban areas (Crude OR and [95% CI]=2.00 [1.24–3.20]; and 1.38 [0.77–2.45], respectively). The major sources of information on HIV/AIDS were associated with comprehensive HIV/AIDS knowledge. Adolescents who reported friends or mass media as their major sources were more likely to have comprehensive HIV/AIDS knowledge compared to those who cited family members as their major source (adjusted OR [95% CI] =1.63 [1.17–2.27] and 1.55 [1.14–2.11], respectively). Adolescents who reported that they had been taught about HIV/AIDS and the related topics at school were 1.59 times more likely to have comprehensive HIV/AIDS knowledge compared to those who did not report being taught on such topics (adjusted OR [95% CI]=1.59 [1.22–2.08]). Discussion on sexual matters with parents or other family members was not associated with comprehensive HIV/AIDS knowledge (adjusted OR [95% CI]=1.01 [0.81–1.25]) (Table 3).

**Table 3:** Logistic regression indicating factors associated with comprehensive HIV/AIDS knowledge among in-school adolescents, Eastern Hararge Zone, Oromia Regional State, Eastern Ethiopia 2011

	Comprehensive knowledge of HIV/AIDS			
			
Variable	Yes	No	Crude OR (95% CI)	Adjusted OR (95% CI)
Family residence				
Rural	494	1407	1	1
Urban	183	682	0.76 (0.63–0.93)	0.84 (0.68–1.03)
Respondent's sex				
Male	542	1443	1	1
Female	**135**	**646**	**0.56 (0.45–0.69)**	**0.60 (0.49–0.75)**
Age group				
<18years	259	882	1	1
≥18years	418	1207	1.18 (1.00–1.41)	1.03 (0.86–1.24)
Family wealth index				
Low	62	275	1	
Middle	**474**	**1480**	**1.42 (1.06–1.91)**	**1.39 (1.03–1.87)**
High	**141**	**334**	**1.87 (1.34–2.63)**	**1.75 (1.24–2.48)**
Major source of HIV/AIDS information				
Family members	109	464	1	1
Teachers	336	1017	1.41 (1.10–1.79)	1.28 (1.00–1.63)
Friends	**91**	**208**	**1.86 (1.35–2.57)**	**1.63 (1.17–2.27)**
Health workers	27	119	1.00 (0.61–1.54)	0.90 (0.56–1.45)
Mass media	**114**	**281**	**1.73 (1.28–2.34)**	**1.55 (1.14–2.11)**
Ever discussed on sexual matter with parents or other family members				
No	194	624	1	1
Yes	270	874	1.00 (081–1.23)	1.01 (0.81–1.25)
Ever been taught HIV/AIDS and sexual matters at school				
No	78	346	1	1
Yes	**599**	**1741**	**1.52 (1.17–1.98)**	**1.59 (1.22–2.08)**

Bold values are to indicate the corresponding *P*-value<0.05.

## Discussion

Only about a quarter of the in-school adolescents had comprehensive HIV/AIDS knowledge. The knowledge was more common among in-school adolescents from families with a middle or higher wealth index, who got HIV/AIDS information mainly from friends or mass media and who received HIV/AIDS and sexual matters education at school. Although the females are highly vulnerable to HIV infection and its effects, they were less likely to have comprehensive HIV/AIDS knowledge compared to males. They were also less likely to have comprehensive pregnancy knowledge, even though they had more knowledge on pregnancy occurrence dates related to the menstrual cycle.

The major source of bias in this study might emerge from the self-administered data collection technique in which respondents might have failed to understand the questions correctly. To overcome this bias, data were collected by a facilitator-guided self-administered method (one facilitator read the questions while respondents worked on their questionnaire and other facilitators monitored whether all the students were progressing at equal pace with the facilitator). The respondents were also provided with questionnaires prepared in all possible languages respondents might understand well. Even though it may be difficult to totally overcome the bias which arises from such methods of data collection, its effect on the findings of this study is negligible.

The level of comprehensive HIV/AIDS knowledge in this study was lower than the previous AIDS awareness and prevention strategy knowledge estimates [2]. The reason might be that, as it has been more than 30 years since the first discovery of AIDS, the awareness should have been evidently high. The comprehensive HIV/AIDS knowledge in this study is slightly lower than the previous prevention strategy knowledge and slightly higher than previous comprehensive knowledge of HIV/AIDS reported by another study [3]. This could be due to the difference in the study populations, as this study was conducted on in-school students while the previous study covered wide population groups.

Comprehensive HIV/AIDS knowledge was associated with the sex of the respondents. The females were less likely to have comprehensive HIV/AIDS knowledge compared to the males. This finding is consistent with the Ethiopian DHS report on HIV prevention strategy knowledge and previous in-school adolescents study which reported low HIV transmission modes knowledge among females [2,4,10]. This may be due to the cultural double standards placed on males and females, which encourage males to discuss HIV/AIDS and related sexual matters issues more openly and discourage or even restrict females from discussing sexual related issues. Similarly, as some cultures in Ethiopia encourage or tolerate male adolescents’ pre-marital sexual intercourse but expect females to remain virgins until marriage, female adolescents will often shy away from discussing sexual issues or refrain from asking questions related to it.

Family wealth index was associated with comprehensive HIV/AIDS knowledge. The adolescents from middle or high family wealth index were more likely to have comprehensive HIV/AIDS knowledge compared to those from a low family wealth index. This is consistent with a finding from another study which reported an increase in mean-knowledge score by increasing socio-economic class [13]. This may be because wealthier families can afford mass media items like televisions, radios, etc. giving their adolescent children access to different HIV/AIDS information sources, particularly as the positive effect was stronger and significant in this study for in-school adolescents whose families reside in rural areas. Furthermore, adolescents from urban families might have different sources of information other than the family-based resources.

Those who cited friends and mass media as their major sources of HIV/AIDS information were more likely to have comprehensive HIV/AIDS knowledge compared to those who reported their parents or other family members as their major sources. This was not consistent with other study findings [6]. This is probably because adolescents may openly discuss more with their friends about sexual matters than with their parents or other family members. This is confirmed by a previous study in Ethiopia [14]. Similarly, mass media may also address such topics more openly in a matter that attracts adolescents’ attention.

Attending classes on HIV/AIDS and sexual matters at school was significantly associated with comprehensive HIV/AIDS knowledge, in that the respondents who reported that they had attended such classes were more likely to have comprehensive HIV/AIDS knowledge compared to those who did not attend such classes. This may be because, even though such topics were integrated into some subjects in schools, some schools and/or teachers may not teach these topics as they might feel they are not well trained on the topic, while some other schools and teachers may teach such topics by making extra effort themselves or by inviting other relevant professionals.

Discussing sexual matters with parents or other family members was not associated with comprehensive HIV/AIDS knowledge. This finding is not consistent with the report of another study [6]. This could be due to the limited knowledge of parents or other family members on HIV/AIDS. Furthermore, what the study participants reported as discussion might not be the open bi-lateral discussion; it might simply be the restrictive order of traditional parents or other family members to make their adolescents stay away from peers and not to listen to sexual related discussion, further limiting their access to other information sources [15].

In conclusion, only about one in four of the in-school adolescents had comprehensive HIV/AIDS knowledge. The factors associated with comprehensive HIV/AIDS knowledge of in-school adolescents were both individual factors (sex) and contextual factors (family wealth index, major source of HIV/AIDS information and ever been taught HIV/AIDS and sexual matters at school). Although the female adolescents are highly vulnerable to HIV infection and its effects, they were by far the less likely to have comprehensive HIV/AIDS knowledge. Thus, HIV/AIDS information, education and communication activities need to be intensified in high schools, including further attention being put on gender, the family wealth disparity, the positive influences of peers, mass media and teaching methods of HIV/AIDS and related issues at schools.
